# Novel Polycaprolactone Based Coating for Catheters: Sustained Antibiotic Release for Enhanced Infection Prevention

**DOI:** 10.3390/ijms26189177

**Published:** 2025-09-19

**Authors:** Kyungmi Kim, Eung Hwi Kim, Seung Zhoo Yoon, Suk Joong Lee

**Affiliations:** 1 Department of Anesthesiology and Pain Medicine, Anam Hospital, College of Medicine, Korea University, Seoul 02841, Republic of Korea; kyungmi_kim@korea.ac.kr; 2 Institute for Healthcare Service Innovation, College of Medicine, Korea University, Seoul 02841, Republic of Korea; b612m45@korea.ac.kr; 3 Department of Chemistry, Korea University, Seoul 02841, Republic of Korea

**Keywords:** polycaprolactone, polymethylhydrosiloxane, ampicillin, sustained release, antibacterial coating, catheter-related infections, biodegradability, medical devices

## Abstract

Healthcare-associated infections are a serious concern, particularly in patients with intravascular catheters. In this study, we developed a novel ampicillin-loaded polycaprolactone (PCL)-coated polyurethane catheter (PUC) wherein polymethylhydrosiloxane (PMHS) enhanced the adhesion between PCL and PUC, ensuring coating durability. PUC were first treated with PMHS, followed by PCL coatings containing 1, 3, or 6 wt% ampicillin. Antibacterial activity against *Listeria innocua* and *Escherichia coli* was evaluated using the plate counting method over 40 d. Scanning electron microscopy confirmed that the coating was uniform and stable for over 40 d. Furthermore, antibacterial efficacy was maintained for 10, 30, and 40 d for the 1, 3, and 6 wt% ampicillin coatings, respectively. Compared to the uncoated controls, the bacterial counts were reduced by over 99.9%. Thus, PMHS pretreatment of a catheter coated with ampicillin-loaded PCL exhibited sustained antibacterial activity. Our findings show PMHS enhances the coating adhesion and ensures gradual uniform degradation of the PCL layer.

## 1. Introduction

Healthcare-associated infections pose a significant challenge to public health owing to their associated high mortality rates [1]. Vascular disease remains a leading cause of death worldwide, driving the increased use of intravascular catheters (ICs) [2], which are indispensable for vascular diagnostics, drug delivery, and surgical interventions. However, despite their critical role in modern medicine, ICs are associated with substantial risks, including catheter-associated urinary tract infections [3,4] and catheter-related bloodstream infections [5,6]. Nosocomial infections affect 4–10% of hospitalized patients in developed countries, with rates as high as 30% in patients in intensive care units [7,8,9]. 

Consequently, prevention of catheter-related infections has become one of the most pressing challenges in healthcare.

IC use is associated with three major complications that significantly affect patient health. Catheter-related bloodstream infections frequently arise from microbial colonization at the puncture site or contamination of the device itself [10], resulting in high mortality rates [11]. Second, thrombus formation on catheter surfaces can lead to detachment of blood clots, which travel through the vascular system and may cause embolism, organ damage, and secondary bloodstream infections due to microbial adhesion [12,13,14]. Third, friction between the catheter and vascular walls can cause endothelial damage, leading to intimal proliferation, embolization, and ischemia, further compromising patient outcomes. These challenges highlight the urgent need for advanced IC designs that incorporate multifunctional surface properties to address these risks effectively [15].

Considering the increasing prevalence of multidrug-resistant infections, coatings with concurrent antibiofilm activity may offer substantial advantages for ICs, provided that they maintain blood compatibility and avoid adverse tissue reactions at the catheter interface. Previous studies have shown that antimicrobial ICs have low hemolytic potential, no cytotoxicity at clinically relevant concentrations, and are functionally compatible with hemostasis [16,17], highlighting the biocompatibility of antibiotic-integrated IC coatings. Clinically meaningful suppression of pathogen colonization and biofilm formation are the core determinants of catheter-related bloodstream infections. Recent advancements have explored the integration of antimicrobial monomers, peptides, and polymer-based coatings to improve antibacterial and antifouling capabilities [18,19,20]. Studies have shown the efficacy of antimicrobial peptide-coated silicone catheters and biofilm-resistant polymer brush coatings [21,22]. However, significant challenges remain, such as coating instability, short-term efficacy, and difficulties in scaling up these technologies for industrial production.

To address these challenges, polymethylhydrosiloxane (PMHS) was introduced to improve the stability and mechanical strength of polycaprolactone (PCL) in polyurethane catheters (PUCs). The biodegradability and biocompatibility of PCL [23,24,25,26,27] enables controlled drug release and low friction with blood streaming, while PMHS enhances adhesion between PCL and PUC, ensuring coating durability. This multifunctional approach offers a promising solution for mitigating catheter-related complications, thereby improving patient safety and clinical outcomes.

Here, we evaluated the efficacy of an antibiotic-releasing catheter with ampicillin embedded within a biodegradable PCL coating on a PUC. We hypothesized that gradual degradation of the PCL matrix would result in sustained antibiotic release, providing a long-term antibacterial environment. To validate this concept, we developed a novel surface modification technique for ICs, utilizing PCL and ampicillin to achieve sustained antibacterial efficacy for over 40 d. Furthermore, we evaluated the antibacterial activity of the PCL-coated catheters against both Gram-positive (*Listeria innocua*) and Gram-negative (*Escherichia coli*) bacteria and compared their performance based on the ampicillin concentration within the PCL coating.

## 2. Results

### 2.1. Chemical Characteristics of PCL-Coated Catheter

**Figure 1:**
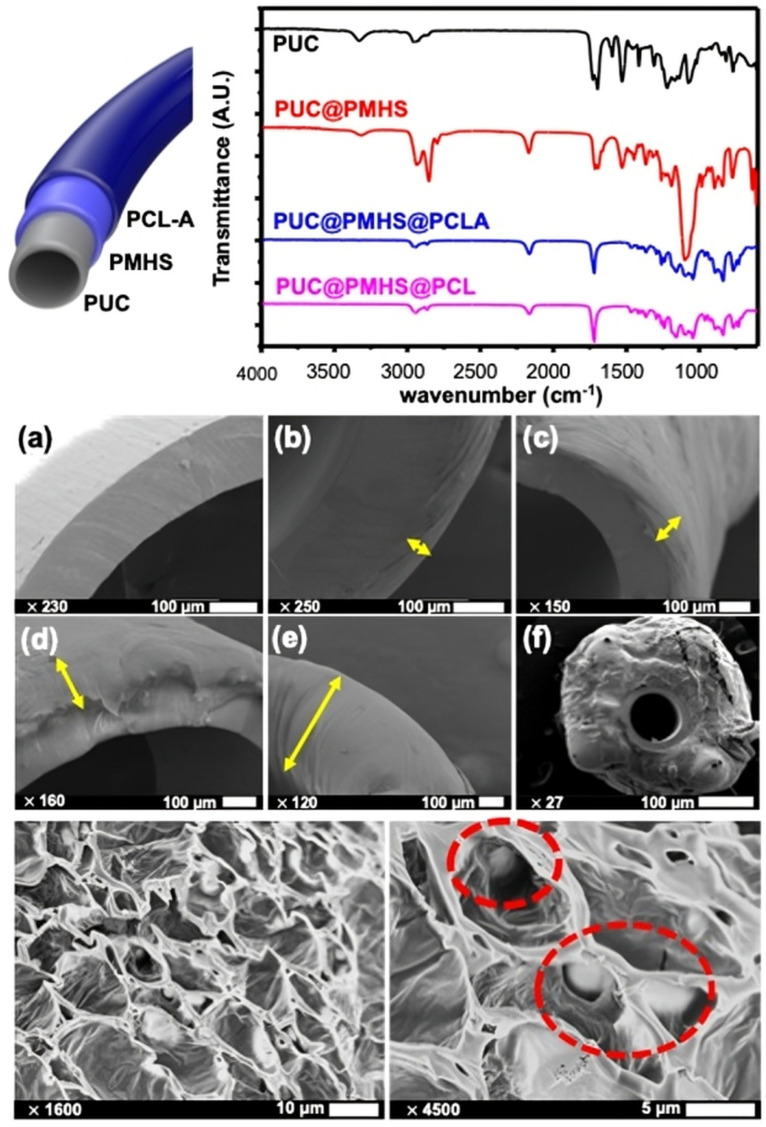
Characterization of PCL-coated catheter. (**Top**) FT-IR spectra of the materials comprising the PCL-coated catheter is shown. (**Middle**) SEM images showing thickness (yellow arrows) after (**a**) 0 (untreated catheter) (**b**) 1 (~50 µm), (**c**) 2 (~100 µm), (**d**) 3 (~150 µm), (**e**) 10 (~500 µm), and (**f**) 20 (~1000 µm) dip cycles. Magnifications and scale bars are indicated on each panel. (**Bottom**) High-magnification SEM of the PCL-A surface showing uniform dispersion of ampicillin (red circles) within the matrix. PCL, polycaprolactone; FT-IR, Fourier-transform infrared; SEM, scanning electron microscopy; PCL-A, a solution of PCL and ampicillin.

We performed Fourier transform infrared (FT-IR) spectroscopy to confirm the sequential assembly of PUC@PMHS@PCL-A (Figure 1, top). The FT-IR spectra of the materials revealed distinct absorption bands corresponding to the respective functional groups. The pristine PUC exhibited the expected urethane bands, including N–H stretching at ~3300 cm^−1^ and carbonyl absorption around 1700–1730 cm^−1^, with amide near 1530–1560 cm^−1^ [28]. After PMHS treatment, new silicone peaks appeared: Si–H stretching at ~2100–2160 cm^−1^, Si–CH_3_ bending at ~1250–1260 cm^−1^, and broad Si–O–Si vibrations between 1000 and 1100 cm^−1^, confirming the presence of the adhesion layer [29]. Following deposition of PCL with ampicillin (PCL-A), strong bands at 1720–1725 cm^−1^ (ester C=O), 1240–1295 cm^−1^ (C–O–C/C–O), and 1160 cm^−1^ (C–O/C–C) verified the PCL over-coating [30]. A weak shoulder at ~1768–1775 cm^−1^ is consistent with the β-lactam carbonyl of ampicillin, supporting drug incorporation [31,32,33].

### 2.2. Physical Characteristics of PCL-Coated Catheter

**Figure 2:**
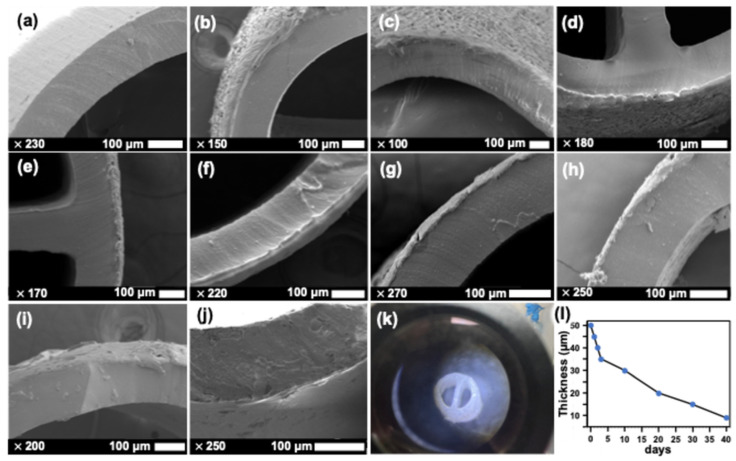
Stability of the PCL layer. (**a**) Cross-sectional SEM of uncoated PUC pretreated with PMHS. A catheter was stored in the dark and gently shaken to emulate a flowing environment (**k**). Cross-sectional SEM at day 0 (~50 µm; (**b**)), 1 (~45 µm; (**c**)), 2 (~40 µm; (**d**)), 3 (~35 µm; (**e**)), 4 (~30 µm; (**f**)), 10 (~30 µm; (**g**)), 20 (~20 µm; (**h**)), 30 (~15 µm; (**i**)), and 40 (~9 µm; (**j**)) shows progressive thinning of the coating. Magnifications and scale bars are shown on each panel. The temporal thinning of the coating is summarized in (**l**). PCL, polycaprolactone; SEM, scanning electron microscopy; PUC, polyurethane catheter; PMHS, polymethylhydrosiloxane.

We used scanning electron microscopy (SEM) analysis to evaluate the surface and cross-sectional morphology of the multilayer catheter (Figure 1, middle and bottom panels). After PMHS treatment and PCL overcoating, the surface appeared continuous and pore-free without interfacial gaps, indicating good adhesion of PCL to PUC via the PMHS interlayer. The cross-sectional views show a monotonic increase in the coating thickness with the number of dip cycles (1, 2, 3, 10, and 20 cycles), consistent with the dip-coating behavior reported for polymer films, with thickness scaling with concentration and repeated deposition. Time-lapse SEM on a single-coat specimen further shows progressive thinning of the PCL layer from ~50 µm (day 0) to ~9 µm (day 40) under storage/shaking, consistent with hydrolytic degradation/erosion of PCL in aqueous conditions described in prior studies (Figure 2). These observations support the intended stable interfacial adhesion (PUC–PMHS–PCL) and controlled, time-dependent reduction in coating thickness, enabling sustained drug release [34,35].

### 2.3. Antibacterial Activity

**Figure 3:**
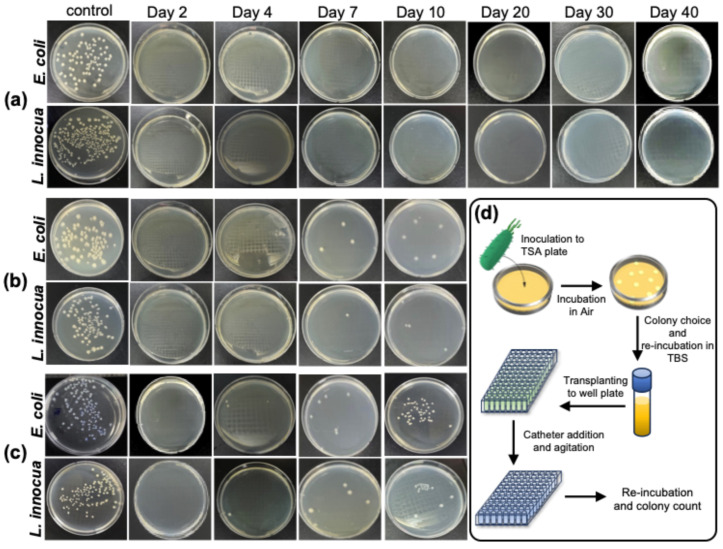
Antibacterial activity of PCL-A coated catheter. Plate-count assays were performed at day 0 and on days 2, 4, 7, 10, 20, 30, and 40. Panels show representative plates for days 2, 4, 7, 10, 20, 30, and 40 for the 6 wt% (**a**), 3 wt% (**b**), and 1 wt% (**c**) ampicillin groups, respectively; CFU counts at all time points are summarized in the text. Schematic illustration of the antimicrobial assay (**d**).

The antibacterial efficacy of the PCL-A-coated catheter was evaluated in vitro against *L. innocua* (Gram-positive) and *E. coli* (Gram-negative) over 40 d using the plate-counting method (Figure 3). The initial bacterial concentration was approximately 10^9^ CFU/mL. Antibacterial activity was assessed at day 0 and on days 2, 4, 7, 10, 20, 30, and 40; Figure 3 shows representative plates for days 2, 4, 7, 10, 20, 30, and 40. The results demonstrated that the PCL-A-coated catheter maintained significant antibacterial efficacy for at least 10 d, regardless of the ampicillin concentration (1, 3, or 6 wt%). Among the tested formulations, the 6 wt% ampicillin-incorporated sample exhibited the highest antibacterial performance, sustaining a controlled release of ampicillin throughout the 40 day period (Figure 3a). Therefore, PCL-A-coated catheter provides long-term antibacterial protection, making it a promising candidate for biomedical applications.

## 3. Discussion

This study describes a novel PCL-coated catheter pretreated with PMHS that shows sustained biodegradability for 40 d. The introduction of PMHS significantly improved the stability of the PCL coating, whereas the gradual degradation of PCL facilitated sustained and controlled ampicillin release. This innovative approach addresses the critical challenges associated with catheter-related infections, coating instability, and short-lived antibacterial effects, positioning this modified catheter as a promising candidate for biomedical applications that require prolonged antimicrobial activity.

A key limitation of conventional antibiotic-coated catheters is the poor adhesion and rapid detachment of the coating layer, which leads to premature drug loss and diminished long-term efficacy [36,37]. Our study demonstrated that PMHS plays significantly stabilized the PCL layer on the PUC surface, ensuring that the coating remained intact for an extended period. Without PMHS, the PCL layers exhibit weak adhesion and rapid detachment from the polyurethane surface upon exposure to physiological conditions, significantly limiting their clinical applicability.

PMHS facilitates strong interfacial bonding between PUC and PCL by forming a robust interconnection structure, thereby enhancing the mechanical stability and adhesion [38,39]. This interfacial stability is essential for ensuring the uniformity of the PCL coating and preventing cracks or delamination over time, compromising drug release kinetics and antibacterial efficacy. SEM analysis confirmed the structural integrity of the coated catheter with gradual but uniform degradation of the PCL layer, indicating the effectiveness of the adhesion-enhancement strategy.

This study developed a catheter with sustained antibiotic release over an extended period. Many traditional antibiotic coatings experience burst release, in which a significant portion of the antimicrobial agent is released in the first few hours or days, leading to short-term effectiveness and an increased risk of bacterial resistance [37,40]. In contrast, our PCL-based biodegradable coating exhibited a well-controlled, sustained release of ampicillin for over 40 d, ensuring prolonged antibacterial activity. The gradual hydrolytic degradation of PCL facilitated the steady and continuous diffusion of ampicillin, preventing the rapid depletion observed in conventional coatings. SEM analysis confirmed that the PCL layer gradually thinned over time, correlating with the antibacterial efficacy tests, demonstrating sustained bacterial inhibition throughout the experimental period. This slow-release mechanism is particularly beneficial for intravascular catheters because it minimizes the risk of biofilm formation, bloodstream infections, and bacterial colonization, which are common challenges in catheter-related infections [41]. The ability to tailor the degradation rate of PCL by adjusting the coating thickness or polymer composition further enhances the versatility of this approach, allowing customization depending on clinical requirements.

To evaluate the antibacterial performance of the modified catheter, plate-counting experiments were conducted on *L. innocua* (Gram-positive) and *E. coli* (Gram-negative) over a 40 d period. The results consistently demonstrated significant bacterial inhibition, with higher ampicillin concentrations (6 wt%) yielding the most prolonged antibacterial activity. Among the tested formulations, 1 wt% ampicillin exhibited effective bacterial inhibition for approximately 10 d, after which bacterial regrowth was observed. The next higher concentration, 3 wt% ampicillin, provided a moderate and sustained antibacterial effect, extending the inhibition for up to 30 d. The highest concentration tested, 6 wt% ampicillin resulted in the most prolonged and consistent antibacterial activity, effectively preventing bacterial colonization for the entire 40 d period. Although the efficacy of ampicillin was not dose-dependent, higher doses showed greater antibacterial effects. Thus, increasing the ampicillin concentration enhances bacterial inhibition without compromising coating stability. These findings highlight the potential clinical advantages of the PCL-coated catheter system. The PCL-based biodegradable matrix offers a controlled and sustained release mechanism, extending the functional lifespan of the catheter, while reducing the risk of antibiotic-resistance development. Furthermore, the biocompatibility and low-friction properties of PCL may contribute to reduced thrombus formation and vascular wall damage, addressing the additional complications associated with IC use.

Although the multifunctional surface modification approach presented in this study demonstrates a promising strategy for next-generation antimicrobial catheters, our study has limitations. All experiments were conducted in a laboratory condition that assumed an environment similar to the human blood stream. Moreover, we arbitrarily set the concentration of the ampicillin without applying the pharmacokinetic model. Future research could focus on further optimizing drug-loading capacities to enhance release kinetics and broaden its application to other medical devices. Additionally, in vivo studies will be essential to confirm biocompatibility, long-term performance, and clinical efficacy.

## 4. Materials and Methods

### 4.1. Preparation of PCL-Coated Catheter

**Figure 4:**
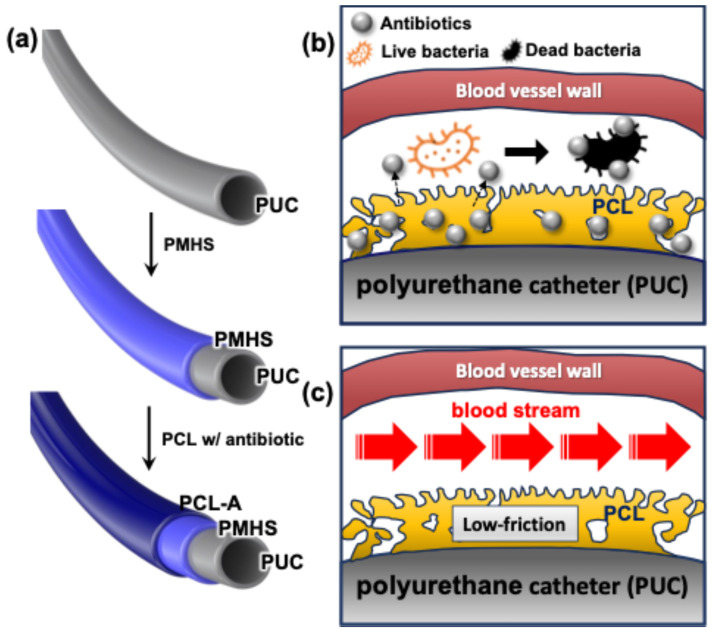
Schematic representation of a PCL-coated catheter. PUC was treated with a solution of PMHS, followed by introducing a solution of PCL-A with varying concentrations of ampicillin (**a**). The mechanism of its antimicrobial performance (**b**), and the low-friction properties provided by the PCL layer (**c**). PCL, polycaprolactone; PUC, polyurethane catheter; PMHS, polymethylhydrosiloxane; PCL-A, a solution of PCL and ampicillin.

The PCL-coated catheter was prepared using a simple dipping method [19]. PUCs were treated with a solution of PMHS, followed by the introduction of a solution of PCL with varying concentrations of ampicillin (Figure 4a).

All reagents and solvents were purchased from commercial sources and used without further purification, unless otherwise indicated. PCL and PMHS were purchased from Sigma-Aldrich (St. Louis, MO, USA) and used without further purification. Commercially available PUCs were used in this study. Ampicillin was purchased from Sigma-Aldrich.

PUC (5 cm in length) was immersed in a PMHS solution prepared by dissolving PMHS (5 g) in ethanol (100 mL) and sonicating in an ultrasonic bath (40 kHz, 200 W) at room temperature for 10 min. The mixture was then incubated at 60 °C for 10 min, followed by gentle shaking at room temperature for 12 h. Then, the catheter was removed and dried at 60 °C for 2 h to yield PUC+PMHS.

First, PCL (0.4 g) was dissolved in dichloromethane (3 mL) and agitated at room temperature for 1 h to achieve complete dissolution. Subsequently, varying amounts of ampicillin (1, 3, and 6 wt% relative to the weight of the polymer) were added to the PCL solution and stirred for an additional 1 h until a homogeneous mixture was obtained (PCL-A solution). PUC+PMHS was immersed in the prepared PCL-A solution until all air bubbles were expelled, which typically required 2 min. The catheter was rotated slowly to ensure uniform coating with the PCL-A solution. After coating, the catheter was removed and dried in an oven at 60 °C for 2 h to obtain a final PCL-coated catheter.

### 4.2. Characteristics of PCL-Coated Catheter

IR spectroscopy was used to analyze the chemical characteristics of the PCL-coated catheters. FT-IR spectra were recorded using an ALPHA FT-IR Spectrometer (Bruker Optics, Billerica, MA, USA) equipped with single-reflection diamond attenuated total reflectance and universal sampling module accessories. Frequencies are determined in cm^−1^. The samples were prepared by thin-film deposition, and the spectra were evaluated to identify the characteristic functional groups and confirm the molecular structure.

To analyze the physical characteristics of the PCL-coated catheter, SEM imaging (JSM-7001F microscope, JEOL, Akishima City, Tokyo, Japan) was conducted at 3 kV, SE detector, and working distance of ~10 mm, after Au sputter coating (~3 nm). Cross-sections were obtained by clean cutting and imaged at low tilt, and the thickness was extracted from calibrated micrographs (instrument scale bar). Images were acquired at low (×27) and high magnifications (×4500). The magnifications and scale bars are mentioned in each figure panel.

To assess the ampicillin embedding within the coating, high-magnification SEM imaging was performed once on a catheter coated with PCL. To measure the coating thickness, the PCL coating was applied to the PUC once, twice, three times, 10 times, and 20 times. The multiple dip-coating layers were an extension of the standard dip-coating technique, which involves the sequential deposition of multiple layers of coatings on a substrate. This method allows the control of the thickness of coatings with tailored properties. The substrate was dipped in the first coating solution, withdrawn after 2 min, and allowed to dry to form the initial coating layer. The coated substrate was then dried to solidify the first layer. This ensured that subsequent layers did not dissolve or blend into the previous layer. The substrate was then sequentially dipped into an additional coating solution. Drying was performed between layers to preserve their integrity. Using this process, the thickness was increased to 1 mm.

To evaluate coating stability, SEM analysis was conducted on a catheter with a single PCL coating. After coating, the catheter was stored in a dark environment, and SEM imaging was performed on days 1, 2, 3, 4, 10, 20, 30, and 40 post-coating to observe changes in the coating thickness over time. It is well known that biodegradability and biocompatibility of hydrophobic PCL enabled controlling the drug release and low friction with blood streaming, while PMHS improved adhesion between PCL and polyurethane (Figure 4b,c). The biodegradability and biocompatibility of the PCL layer in the PCL-coated catheter were investigated by immersing the catheter in phosphate-buffered saline solution with gentle shaking.

### 4.3. Antibacterial Activity Study

The antibacterial properties of the prepared materials (single PCL coating, 1, 3, or 6 wt% ampicillin) were tested against *L. innocua* and *E. coli* on days 0, 2, 4, 7, 10, 20, 30, and 40. Bacterial cultures were subcultured twice in Tryptic Soy Broth medium at 37 °C for 24 h. The cultures were centrifuged at 3500 rpm for 10 min and washed twice with sterile diluted phosphate-buffered saline. The initial bacterial concentration was ~10^9^ CFU/mL. Prepared catheters were placed in 24-well plates, and 50 μL of bacterial suspension was added to each catheter surface. The contaminated catheters were incubated in the dark for 20 min, followed by incubation at 60 rpm for 2 h. After dilution (×10^5^), 20 μL aliquots were spread onto Tryptic Soy Agar plates and incubated at 37 °C for 24 h. Colony counts were recorded, and each experimental condition was tested in triplicate.

## 5. Conclusions

After pretreatment with PMHS, a catheter coated with ampicillin-loaded PCL showed sustained antibacterial activity over 40 d. The PMHS enhanced the coating adhesion between PUC and PCL-A and ensured gradual but uniform degradation of the PCL layer and sustained release of ampicillin. This novel catheter may overcome the limitations of conventional coatings, particularly the poor adhesion and rapid delamination of the coating layer, resulting in premature drug loss and reduced long-term efficacy. This technology has the potential to improve patient safety, reduce catheter-related infections, and enhance the longevity of implanted medical devices.

## Data Availability

No new data were created or analyzed in this study. Data sharing is not applicable to this article.

## Abbreviations

The following abbreviations are used in this manuscript:

IC	intravascular catheters
FT-IR	Fourier-transform infrared
PCL	polycaprolactone
PCL-A	a solution of PCL with ampicillin
PMHS	polymethylhydrosiloxane
PUC	polyurethane catheter
SEM	scanning electron microscopy
